# The Influence of Oxygen Concentration during MAX Phases (Ti_3_AlC_2_) Preparation on the α-Al_2_O_3_ Microparticles Content and Specific Surface Area of Multilayered MXenes (Ti_3_C_2_T_x_)

**DOI:** 10.3390/ma12030353

**Published:** 2019-01-23

**Authors:** Błażej Scheibe, Vojtech Kupka, Barbara Peplińska, Marcin Jarek, Krzysztof Tadyszak

**Affiliations:** 1 NanoBioMedical Centre, Adam Mickiewicz University, 61 614 Poznań, Poland; barbara.peplinska@amu.edu.pl (B.P.); marcin.j@amu.edu.pl (M.J.); tadyszak@amu.edu.pl (K.T.); 2 Regional Centre for Advanced Technologies and Materials, Department of Physical Chemistry, Faculty of Science, Palacky University Olomouc, 771 46 Olomouc, Czech Republic; vojtech.kupka@gmail.com

**Keywords:** MAX phases, Ti_3_AlC_2_, MXenes, Ti_3_C_2_T_x_, α-Al_2_O_3_ particles, porosity

## Abstract

The high specific surface area of multilayered two-dimensional carbides called MXenes, is a critical feature for their use in energy storage systems, especially supercapacitors. Therefore, the possibility of controlling this parameter is highly desired. This work presents the results of the influence of oxygen concentration during Ti_3_AlC_2_ ternary carbide—MAX phase preparation on α-Al_2_O_3_ particles content, and thus the porosity and specific surface area of the Ti_3_C_2_T_x_ MXenes. In this research, three different Ti_3_AlC_2_ samples were prepared, based on TiC-Ti_2_AlC powder mixtures, which were conditioned and cold pressed in argon, air and oxygen filled glove-boxes. As-prepared pellets were sintered, ground, sieved and etched using hydrofluoric acid. The MAX phase and MXene samples were analyzed using scanning electron microscopy and X-ray diffraction. The influence of the oxygen concentration on the MXene structures was confirmed by Brunauer-Emmett-Teller surface area determination. It was found that oxygen concentration plays an important role in the formation of α-Al_2_O_3_ inclusions between MAX phase layers. The mortar grinding of the MAX phase powder and subsequent MXene fabrication process released the α-Al_2_O_3_ impurities, which led to the formation of the porous MXene structures. However, some non-porous α-Al_2_O_3_ particles remained inside the MXene structures. Those particles were found ingrown and irremovable, and thus decreased the MXene specific surface area.

## 1. Introduction

Since the discovery of graphene [1], the scientific trend in nanomaterial sciences turned toward two-dimensional (2D) nanostructures. One of the most intensively investigated groups of 2D nanostructures are transition metal carbides, nitrides and carbonitrides called MXenes [2,3]. MXenes are derived from MAX phases, layered and hexagonal nanolaminates named for their general formula—M_n+1_AX_n_. The M is a transition metal, the A is an A group (mostly IIIA and IVA) element and the X is a C and/or N. The n parameter (n = 1, 2 or 3) determines the formation of the 211, 312 or 413 structures [4].The removal of the A layer via wet chemistry methods leads to the formation of MXenes (M_n+1_X_n_T_x_)—multilayered accordion-like structures, where T_x_ refers to Ti-bonded -F, -OH or -O functional groups [5]. The first obtained MXene was Ti_3_C_2_T_x_, derived from a Ti_3_AlC_2_ MAX phase via Al layer removal [6]. Later, the delamination of Ti_3_C_2_T_x_ toward single-layers led to an increase in the specific surface area (SSA) and allowed for one of the highest volumetric capacitances (520 F/cm^3^ at 2 mV/s), which exposed them as a potential candidate for electrical double layer capacitors [7]. Thanks to their lamellar structure, conductive core and hydrophilic surface, MXenes can host many different cations between their layers. Thus, they can be widely applied in different energy storage devices, such as in Na-ions [8] and Li-S batteries [9], supercapacitors [10] or other branches of science like adsorption [11] and catalysis [12]. Currently, the SSA of the multilayered MXenes is controlled at the post-formation stage via different approaches: (i) increasing the distance between layers via intercalation with ions or guest molecules [13], (ii) physical adsorption or covalent linkage with guest molecules or nanoparticles [14], and (iii) formation of composites with other 2D nanomaterials [15]. In our work we took a totally different approach and investigated the possibility of controlling the porosity and specific surface area of multilayered Ti_3_C_2_T_x_ MXenes at the pre-formation stage—during the preparation of the Ti_3_AlC_2_ MAX phase. The accordion-like MXene structure is held together by hydrogen bonds between functional groups of individual graphene-like MXene sheets [16]. However, our observations indicated that the multilayered structures are also stable due to presence of the α-Al_2_O_3_ particles ingrown inside MXene microparticles. We deduced that the α-Al_2_O_3_ particles are formed during MAX phase sintering, depending on the oxygen concentration. This process is similar to the formation of TiC impurities in the MAX phase matrix, where the non-stoichiometric composition of the starting powders leads to the occurrence of carbon excess or aluminum deficiency during the sintering of the Ti-Al-C based MAX phases [17]. In order to prove our hypothesis, we prepared three different Ti_3_AlC_2_ samples, where TiC-Ti_2_AlC powder mixtures were conditioned and cold pressed into pellets in argon, air and oxygen environments. Both Ti_3_AlC_2_ and Ti_3_C_2_T_x_ samples were investigated by means of scanning electron microscopy (SEM), X-ray diffraction (XRD) and physisorption analyses. As expected, the environmental oxygen concentration during MAX phase preparation had a significant influence on the amount of Al_2_O_3_ impurities in different MXene samples, and thus on their porosity and specific surface area.

## 2. Materials and Methods

### 2.1. Materials

All the reagents were of analytical grade and used without further purification. Absolute ethanol (99.8%) was purchased from POCH. Hydrofluoric acid (HF) (48%), phosphoric acid (≥99%) and titanium carbide (98%) (325 mesh) were obtained from Sigma-Aldrich. Maxthal 211—Ti_2_AlC (325 mesh) was purchased from Kanthal (Hallstahammar, Sweden). The N_2_ (5.0), Ar (5.0) and O_2_ (5.0) were purchased from Linde (Kraków, Poland). All solutions were prepared on the basis of MilliQ (13.6 MΩ/cm) type 1 water (T1-H_2_O).

### 2.2. MAX Phase and MXene Synthesis

The MXene samples were obtained from Ti_3_AlC_2_ MAX sinters prepared under different conditions. Briefly, three mixtures of Ti_2_AlC and TiC powders (1:1 molar ratio) were weighed in air and mixed by ball milling in air for 12 h (agate grinding jars and balls). Each powder mixture was conditioned in a glove-box for 24 h in a different gas environment: (1) argon, (2) air or (3) oxygen, and subsequently cold pressed (10 tons) into 13 mm pellets inside the glove-box. The as-prepared pellets were transferred from the glovebox to a horizontal tube furnace. Ti_3_AlC_2_ MAX phases were prepared via the volume combustion synthesis of pellets at 1350 °C (ramp 10 °C/min) in Ar flow for 2 h. The pellets were ground in 99.5% alumina mortar and sieved through a 400 mesh sieve. As-prepared Ti_3_AlC_2_ microparticle powders (<37 µm) were labeled Ti_3_AlC_2_-Ar, Ti_3_AlC_2_-Air and Ti_3_AlC_2_-O_2_ according to the preparation conditions. Three MAX phase powders underwent aluminum layer etching with hydrofluoric acid (2 g Ti_3_AlC_2_/20 mL HF) in plastic jars at 40°C for 24 h under continuous stirring. As-obtained Ti_3_C_2_T_x_-Ar, Ti_3_C_2_T_x_-Air and Ti_3_C_2_T_x_-O_2_ microparticles were purified through cycles of washing with T1-H_2_O and centrifugation 24.000 rpm for 5 min, until the supernatant reached a pH of 6.5. All purified samples were dispersed in absolute ethanol and left overnight on a Petri dish to evaporate in the oven at 80 °C for further characterization.

### 2.3. Characterization

The MAX phase and MXene samples morphology was investigated by scanning electron microscopy (SEM) (JEM-7001TTLS, JEOL, Akishima, Japan). Powder X-ray diffraction (XRD) studies of Ti_3_AlC_2_ and Ti_3_C_2_T_x_ were carried out on an Empyrean (PANalytical, Royston, UK) diffractometer using Cu Kα radiation (1.54 Å), reflection-transmission spinner (sample stage) and PIXcel3D detector. Surface area and pore size analyses of MXenes and MAX powders were performed by means of N_2_ adsorption-desorption measurements at −196.15 °C on a volumetric gas adsorption analyzer (3Flex, Micromeritics, Norcross, GA, USA) up to 0.965 P/P_0_. Prior to the analysis, the samples were degassed in a vacuum (7 × 10^−2^ mbar) for 12 h at 130 °C, while high purity (99.999%) N_2_ and He gases were used for the measurements. The Brunauer-Emmett-Teller area (BET) was determined with respect to Rouquerol criteria for BET determination in the range of 0.1–0.3 P/P_0_, assuming a molecular cross-sectional area of 16.2 Å^2^ for N_2_. The isotherms were further analyzed for pore size calculation using the Barret-Joyner-Halenda (BJH) method. An average slit-pore width was calculated according to the formula:

(1) $$\bar{w_{p}}=2V/S_{BET}$$

where $\bar{w_{p}}$ is an average slit-pore width, *V* is the total pore volume and *S_BET_* is the surface area [18].

## 3. Results and Discussion

**Figure 1:**
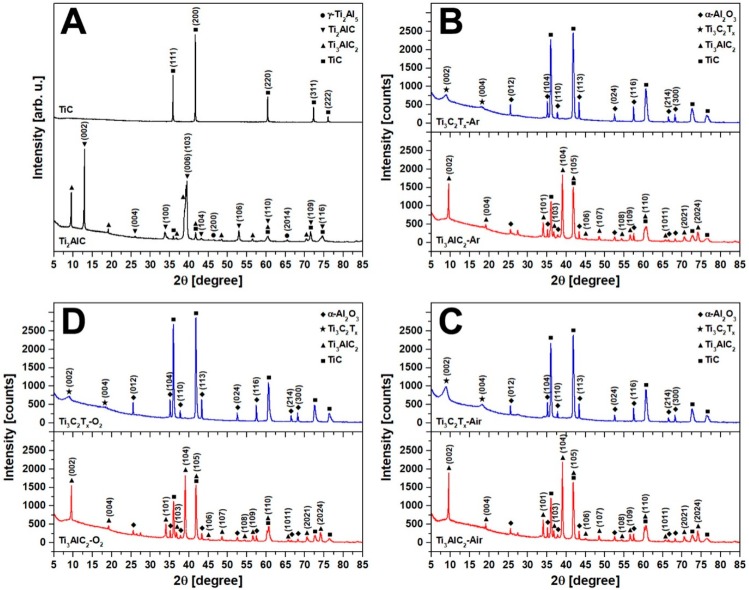
The XRD patterns of (**A**) TiC/Ti_2_AlC powders as well as Ti_3_AlC_2_/Ti_3_C_2_T_x_ obtained by powder mixtures prepared in (**B**) argon, (**C**) air and (**D**) oxygen environments.

X-ray diffraction is an essential technique proving the successful synthesis of Ti_3_AlC_2_ MAX phases and the formation of Ti_3_C_2_T_x_ MXenes. Figure 1 presents XRD patterns of powder precursors (TiC/Ti_2_AlC) (Figure 1A) and all prepared samples (Ti_3_AlC_2_/Ti_3_C_2_T_x_) (Figure 1B–D). The TiC XRD pattern exhibits five diffraction peaks at 2θ = 35.9° (111), 41.73° (200), 60.44° (220), 72.34° (311) and 76.11° (222), which correspond to pure face-centered cubic TiC phase [19]. The commercial Ti_2_AlC powder exhibits nine 211 MAX phase specific peaks at 2θ = 12.95° (002), 26.08° (004), 33.80° (100), 39.52° (103), 43.17° (104), 52.97° (106), 60.53° (110), 71.55° (109) and 74.61° (116). The highly intense (002) peak indicates a high degree of crystallinity of the material. The Ti_2_AlC powder is contaminated to some extent with Ti_3_AlC_2_, TiC and γ-Ti_2_Al_5_ particles, which presence is confirmed in the XRD spectrum [20]. Regardless of the environment in which TiC and Ti_2_AlC powder mixtures were conditioned and cold pressed, the sintering of pellets led to the formation of the Ti_3_AlC_2_. All Ti_3_AlC_2_ samples presented similar XRD patterns and shown fourteen 312 MAX phase peaks at 2θ = 9.57° (002), 19.2° (004), 34.05° (101), 36.79° (103), 39.05° (104), 41.86° (105), 44.99° (106), 48.55° (107), 54.34° (108), 56.57° (109), 60.45° (110), 65.64° (1011), 70.57° (2021) and 74.14° (2024) [21]. The position of the peaks was almost identical with non-significant shifts. The only difference was observed in the intensities of the (002), (004) and (103) peaks. No peaks related to the 211 Ti_2_AlC phase were observed. However, the Ti_3_AlC_2_ XRD patterns exhibited five TiC-related peaks: (111), (200), (220), (311), (222) as well as eight peaks at 2θ = 25.56° (012), 35.14° (104), 37.76° (110), 43.33° (113), 52.53° (024), 57.48° (116), 66.50° (214) and 68.19° (300), which corresponded to the α-Al_2_O_3_ particles [22]. The removal of the Al layer from the MAX phases led to disappearance of all the Ti_3_AlC_2_ related peaks and shift of the (002) and (004) peaks toward lower values from 2θ = 9.57° and 19.19° to 2θ = 8.86° and 18.04°, respectively. In general, the shift and broadening of the (002) peak indicated the successful formation of the MXenes [23]. The Ti_3_C_2_T_x_ XRD patterns also exhibited peaks related to TiC and α-Al_2_O_3_, both of which are well known contaminants of multilayered MXene structures [24]. The TiC and α-Al_2_O_3_ particles were formed during the Ti_3_AlC_2_ sintering and remained as impurities after Al removal [25]. Interestingly, the TiC and α-Al_2_O_3_ related peaks became sharper and their intensities increased after aluminum etching. The highest increase was observed for the Ti_3_C_2_T_x_-O_2_ and it was not related to the preferred orientation of the MXene powder on the Si holder but rather to oxygen availability during the sintering. This observation could be explained by the following:

(1) increased O_2_ concentration led to increased formation of Al_2_O_3_;
(2) increased Al_2_O_3_ formation led to a decreased availability of Al atoms for Ti_3_AlC_2_ synthesis;
(3) decreased Al atoms content led to increased formation of TiC due to stoichiometry disturbance.

**Figure 2:**
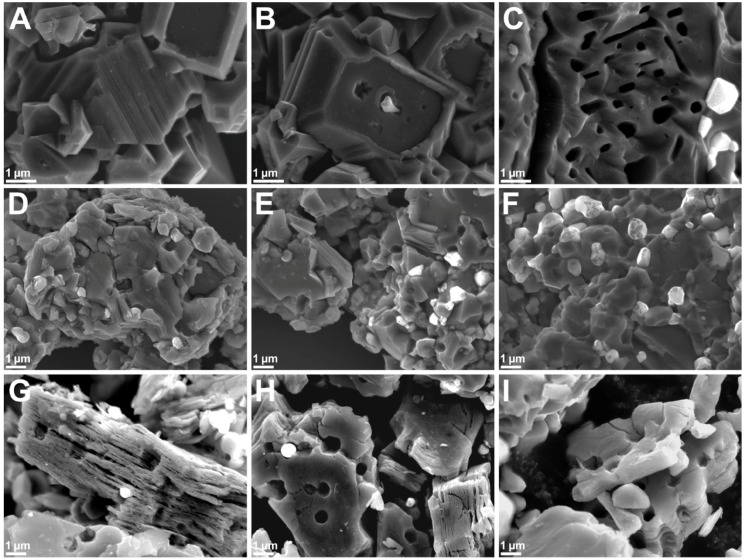
SEM micrographs of MAX phase pellets (**A**–**C**), ground powders (**D**–**F**) and purified MXene particles (**G**–**I**) prepared in Ar (**A**,**D**,**G**), Air (**B**,**E**,**H**) and O_2_ (**C**,**F**,**I**) environments.

The morphologies of the Ti_3_AlC_2_ and Ti_3_C_2_T_x_ samples prepared in different environments are shown in Figure 2. All presented micrographs are representative of the majority of each sample. The top, middle and bottom rows correspond to: (i) lightly ground MAX phase pellets (Figure 2A–C), (ii) sieved MAX phase powders (Figure 2D–F), and (iii) purified MXene powders (Figure 2G–I), respectively. The left, middle and right columns are related to the samples prepared by powder mixtures conditioned in Ar, air and O_2_ environments, respectively. From Figure 2A–C one can see that regardless of the applied gas, MAX phase pellets possessed a layered structure. The only difference was the amount of nonconductive α-Al_2_O_3_ particles (shining white due to charge accumulation) or the number of holes after they fell out. All investigated samples were contaminated with alumina particles, which is in agreement with the XRD analyses. The morphology of Ti_3_AlC_2_-Ar and Ti_3_AlC_2_-Air was similar. The α-Al_2_O_3_ particles presence in the Ti_3_AlC_2_-Ar samples was unexpected. At first we suspected oxygen molecules adsorbed onto the cold-pressed pellet’s surface during its exposition to environmental air while being transferred from the glovebox to the horizontal furnace. The evaporating Al in contact with adsorbed O_2_ molecules formed an Al_2_O_3_ layer on sintered pellet surface. However, the morphology is quite different because the Al_2_O_3_ layer is made of particles in the form of rods and needles (Figure S1). Therefore, we believe that the formation of α-Al_2_O_3_ particles in the Ti_3_AlC_2_ matrix was caused by the availability of atmospheric oxygen during the weighing and mixing of the powders. The 24 h conditioning of the powder mixture in Ar did not lead to desorption of the O_2_ molecules. Aluminum has a high affinity to O_2_ so in order to prepare perfect Ti_3_AlC_2_ MAX phases, all preparation steps should be performed in an Ar filled glovebox [26].

**Figure 3:**
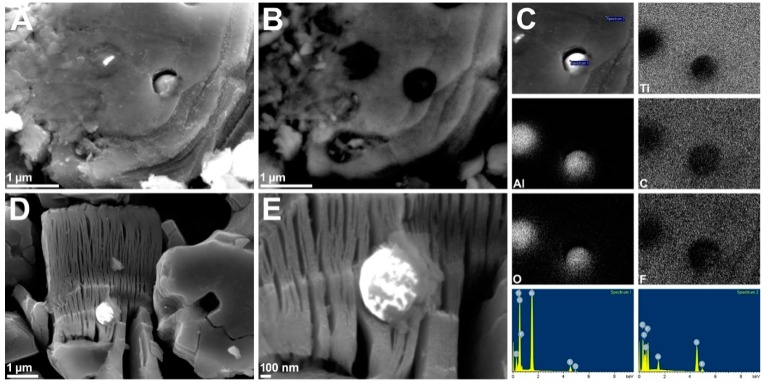
SEM micrographs of Ti_3_C_2_T_x_-Air in SEI (**A**,**D**,**E**), COMPO (**B**) modes, and EDS analyses (**C**).

In the case of Ti_3_AlC_2_-O_2_ (Figure 2F) one can observe a plethora of alumina particles. Both α-Al_2_O_3_ and holes from which the particles fell are present in the derived multilayered MXenes (Figure 2G–I). We tried to remove α-Al_2_O_3_ particles using H_3_PO_4_ solutions in a concentration range of 5% to 95%, as well as etchant mixtures for the removal of thin layers of Al_2_O_3_, but without success. Then, we tried to remove α-Al_2_O_3_ mechanically by stirring, shaking, sieving and exploiting the difference in solubility via multiple cycles of dispersing/centrifugation, but with little success. We performed comprehensive SEM analyses of MXene structures comparing micrographs captured in classic SEI (secondary electron imaging) and COMPO (backscattering electrons) modes as well as with EDS mapping and elemental analysis (Figure 3). The application of COMPO mode (Figure 3B) allowed us to distinguish the lighter and heavier atoms in the SEM image. Lighter elements appeared darker, while heavier appeared lighter. This mode was used to distinguish MXene structures from α-Al_2_O_3_ particles and to reveal the position of alumina particles in the structure. Comparing Figure 3A,B, one can see the alumina particles that are hidden in the MXene structure beneath the top Ti_3_C_2_T_x_ layers. This observation proves that they were formed during Ti_3_AlC_2_ sintering and were not introduced to the MAX phase structure by powder grinding in alumina mortar or from the remains of the Al_2_O_3_ layer that was mechanically removed from Ti_3_AlC_2_ pellet surface. The presence of α-Al_2_O_3_ particles was also confirmed by EDS mapping and analyses performed on top of the microparticle and MXene (Figure 3C). The Al and O distribution maps clearly describe the α-Al_2_O_3_ position in the MXene sample. Spectrum 1 of the α-Al_2_O_3_ exhibited intense Al (18.83% atomic—at.) and O (59.03% at.) peaks at Kα = 1.486 keV and Kα = 0.525 keV, respectively. The intensities of the peaks related to Ti (3.35% at.), C (14.70% at.) and F (4.09% at.) at Kα = 4.508/Lα = 0.452 keV, Kα = 0.277 keV and Kα = 0.677 keV, respectively, were low and could be assigned to some MXene fragments remaining on the α-Al_2_O_3_ surface. In the case of Spectrum 2, the intensities of Al (1.78% at.) and O (18.21% at.) peaks were low, while MXene peaks: Ti (20.12% at.), C (27.69% at.) and F (32.20% at.) dominated. The presence of O atoms confirmed the existence of the MXene terminal groups, while the Al presence could have been related to the surface adsorbed AlF_3_ impurities. Profound SEM analysis of the Ti_3_C_2_T_x_-Air sample revealed that α-Al_2_O_3_ particles were not only hidden in the MXene structure but were grew in and covered by MXenes (Figure 3D,E), proving again that they were formed during MAX phase sintering. This is the reason why α-Al_2_O_3_ impurities are so hard to remove and why it is important to control the oxygen concentration during MAX phase preparation. This led us to conclude that multilayered MXenes are not only held together by the hydrogen bonds between Ti_3_C_2_T_x_ monolayers, but also grown in α-Al_2_O_3_ particles. The majority of the alumina particles possessed a spherical shape. However, some of them grew in a triangular or tooth-like shape (Figure S2). This observation indicates that the α-Al_2_O_3_ impurities formed during MAX phase sintering adopted their shape to the limited space available between the forming Ti_3_AlC_2_ layers.

**Figure 4:**
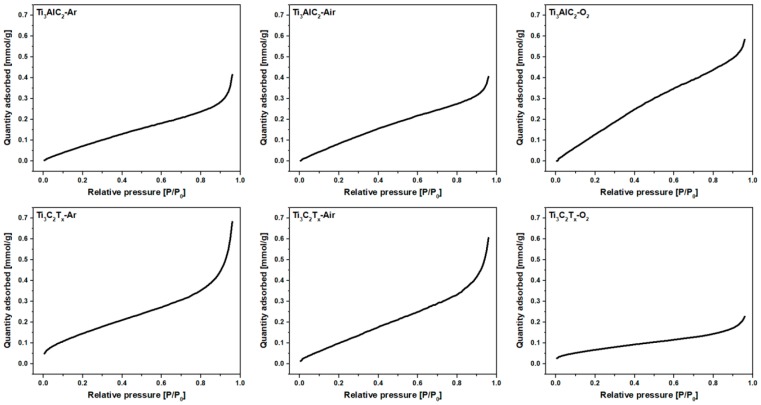
The N_2_ adsorption isotherms of MAX phase and MXene powders.

The specific surface area, pore volume and pore size distribution of the MAX phases and derived MXenes were determined from physisorption measurements. The N_2_ adsorption isotherms are shown in Figure 4.

According to International Union of Pure and Applied Chemistry (IUPAC) classification, all isotherms corresponded to the Type II isotherm, which is typical for macroporous solids [27]. The calculated specific surface area, pore volume and pore diameters are presented in Table 1.

**Figure 5:**
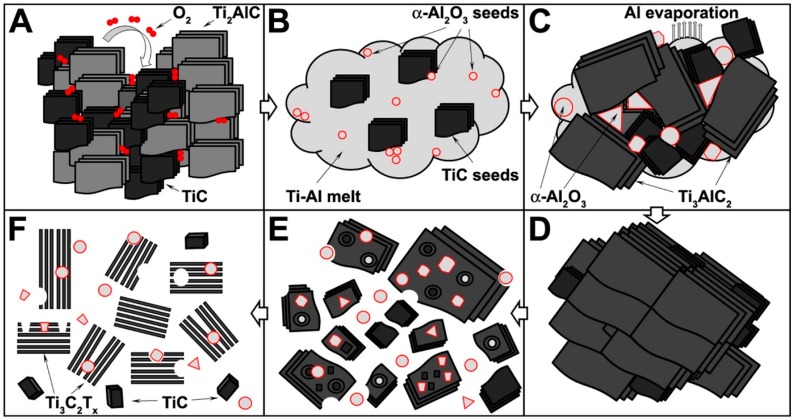
The formation of α-Al_2_O_3_ microparticles during Ti_3_AlC_2_ MAX phase synthesis.

In the case of MAX phase powders, the Ti_3_AlC_2_-Ar and Ti_3_AlC_2_-O_2_ samples shown the highest and the lowest SSA, respectively. This effect is related to the concentration of holes remaining in the Ti_3_AlC_2_ structure after mechanical removal of the α-Al_2_O_3_ particles during the grinding process, what was previously observed in SEM micrographs (Figure 2A,C). According to the different scientific reports, the SSA of multilayered Ti_3_C_2_T_x_ is in the range of 5 to 90 m^2^/g, which is related to the (i) preparation method [28], (ii) state of oxidation/decomposition [29], and (iii) intercalated ions or guest molecules [30,31,32]. The SSA values of the investigated samples were typical for multilayered MXenes. For the Ti_3_C_2_T_x_-Ar and Ti_3_C_2_T_x_-Air samples, the SSA values were higher in comparison with the parental MAX phases, which was due to the removal of the Al atoms after HF etching. All the MXene samples were contaminated by various amounts of TiC and α-Al_2_O_3_ impurities. Based on the XRD and SEM analyses, the highest concentration of non-porous particles was expected for the Ti_3_C_2_T_x_-O_2_ sample. This assumption was fully confirmed by the two-fold lower SSA value compared with the other investigated MXene samples, and by almost a three-fold lower pore volume in comparison with Ti_3_C_2_T_x_-Ar. The physisorption measurements of the MAX phase and MXene powders clearly shown the influence of high oxygen concentration, during pellet preparation, on the formation of non-porous impurities in MAX phases, and thus MXenes porosity. Based on the information obtained through XRD analyses, SEM-EDS investigations and physisorption measurements, the formation of α-Al_2_O_3_ particles in the Ti_3_AlC_2_ matrix as well as their fate after conversion of the MAX phases to MXene structures is presented schematically in Figure 5.

At first, the oxygen molecules are adsorb on the TiC/Ti_2_AlC particle surface and remain there during pellet preparation (Figure 5A). Next, Ti-Al melt forms at the early stages of the sintering process. The adsorbed O_2_ molecules form the initial α-Al_2_O_3_ seeds with Al atoms released from the Ti_2_AlC MAX phase. The decomposition of Ti_2_AlC also leads to the formation of TiC grains (Figure 5B). When the temperature reaches 1350 °C, the following events occur: (i) Ti_3_AlC_2_ layers start to precipitate from Ti-Al melt, (ii) the Al atoms evaporate, (iii) the α-Al_2_O_3_ grains grow between Ti_3_AlC_2_ structures, and (iv) the TiC particles grow due to an insufficient amount of Al atoms for MAX phase formation (Figure 5C). When the process is finished, the polycrystalline Ti_3_AlC_2_ sinter is obtained (Figure 5D). The mortar grinding/ball milling treatments break the pellet structure, decrease the size of the particles and release α-Al_2_O_3_ and TiC impurities. Part of the ingrown alumina particles remain embedded in the Ti_3_AlC_2_ matrix (Figure 5E). The aluminum atoms are removed from the MAX phase by hydrofluoric acid treatment, which lead to the formation of the multilayered MXene structure. The HF acid has no influence onto TiC or α-Al_2_O_3_ particles, thus they are present as an impurity in the Ti_3_C_2_T_x_ sample (Figure 5F).

## 4. Conclusions

We investigated and explained the influence of oxygen concentration during MAX phase preparation on the formation of alumina particles during MAX phase sintering, and thus on the properties of derived MXenes. High concentrations of O_2_ molecules adsorbed on TiC/Ti_2_AlC particles or inside the cold-pressed pellet led to the increased formation of α-Al_2_O_3_ particles in the Ti_3_AlC_2_ matrix. The hydrofluoric acid treatment led to the formation of multilayered Ti_3_C_2_T_x_ featured by the presence of ingrown alumina particles and a plethora of structural holes created after the particles fell out. Ti_3_C_2_T_x_ obtained by the conditioning of TiC-Ti_2_AlC powders in an O_2_ environment possessed two-fold lower specific surface area in comparison with MXenes obtained by the conditioning of powders mixture in the Ar-filled glovebox. Therefore, in order to prepare high quality MXenes, oxygen should be avoided at each step of preparation—starting from weighing of the powders for Ti_3_AlC_2_ sintering and going all the way to the removal of O_2_ molecules adsorbed on the MAX phase pellet surface before sintering. 

The presence of large holes in the Ti_3_C_2_T_x_ structure brings an opportunity to fill them with i.e., magnetic or catalytically active particles, or bioactive molecules, which could broaden potential application of multilayered MXenes toward environmental remediation, catalysis or drug delivery systems. The only issue is the plethora of remaining α-Al_2_O_3_ impurities, which are hard to remove due to the natural resistance to wet chemistry methods and emplacement in the MXene structure. We believe that applying intercalation/delamination procedures could lead to the formation of tiny, hydrophilic Ti_3_C_2_T_x_ monolayers due to the already fragmentized multilayered structure. Then, as-prepared MXenes could be separated from the insoluble α-Al_2_O_3_ particles by several simple washing-centrifugation cycles. 

Currently, all experimental approaches related to preparation of tiny monolayers are based on: (i) extensive mechanical grinding and ball milling of the MAX phases, or (ii) ultrasound treatment of the multilayer MXenes, to delaminate and cut MXene layers into pieces. The former approach can result in severe damage to the MXene flakes after the etching process, and later can lead to the formation of structural defects and MXene decomposition. Actually, we believe that the preparation of multilayered MXenes with a plethora of structural holes could be a novel approach and also an efficient route for obtaining small MXene monolayers without the risk of defects formation or decomposition of the structure.

## Figures and Tables

**Table 1:** Specific surface area, pore volume and pore diameters calculated for investigated MAX phases and derived MXenes.

Sample	Specific Surface Area (m^2^/g)	Pore Volume (cm^3^/g)	Average Pore Width (BJH) (nm)	Average Slit-Pore Width (nm)
Ti_3_AlC_2_-Ar	10.57	0.0133	6.58	2.5
Ti_3_AlC_2_-Air	12.95	0.0134	5.51	2.1
Ti_3_AlC_2_-O_2_	22.46	0.0195	5.12	1.7
Ti_3_C_2_T_x_-Ar	13.70	0.0219	6.86	3.2
Ti_3_C_2_T_x_-Air	13.64	0.0196	6.30	2.9
Ti_3_C_2_T_x_-O_2_	5.96	0.0075	5.38	2.5

## Supplementary Materials

The following are available online at http://www.mdpi.com/1996-1944/12/3/353/s1. Figure S1—The SEM micrographs (A–E) and EDS analysis (F) of the Al_2_O_3_ layer scratched from the Ti_3_AlC_2_ pellets and deposited on the carbon tape. Figure S2—The SEM micrographs of Ti_3_AlC_2_-Air (A,C) and Ti_3_AlC_2_-Ar (B,D), lightly broken pellets presenting triangle-shaped α-Al_2_O_3_ nanoparticles (C) and highly symmetrical leftover holes (D).
