# Supplementary material for: The Influence of Oxygen Concentration during MAX Phases (Ti3AlC2) Preparation on the α-Al2O3 Microparticles Content and Specific Surface Area of Multilayered MXenes (Ti3C2Tx)
Source: Materials (Basel). 2019 Jan 23;12(3):353. doi: 10.3390/ma12030353 (PMC6384598; doi:10.3390/ma12030353)
Supplement: Supplementary file 1 [file materials-12-00353-s001.pdf]

Article – Supporting Information

The Influence of Oxygen Concentration during MAX Phases (Ti_3AlC_2) Preparation on the α-Al_2O_3 Microparticles Content and Specific Surface Area of Multilayered MXenes ($Ti_3C_2T_x$)

Błażej Scheibe[1,*], Vojtech Kupka[2], Barbara Peplińska[1], Marcin Jarek[1] and Krzysztof Tadyszak[1]

1 NanoBioMedical Centre, Adam Mickiewicz University, 61 614 Poznań, Poland; barbara.peplinska@amu.edu.pl (B.P.); marcin.j@amu.edu.pl (M.J.); tadyszak@amu.edu.pl (K.T.)

2 Regional Centre for Advanced Technologies and Materials, Department of Physical Chemistry, Faculty of Science, Palacky University Olomouc, 771 46 Olomouc, Czech Republic; vojtech.kupka@gmail.com

* Correspondence: bscheibe@amu.edu.pl

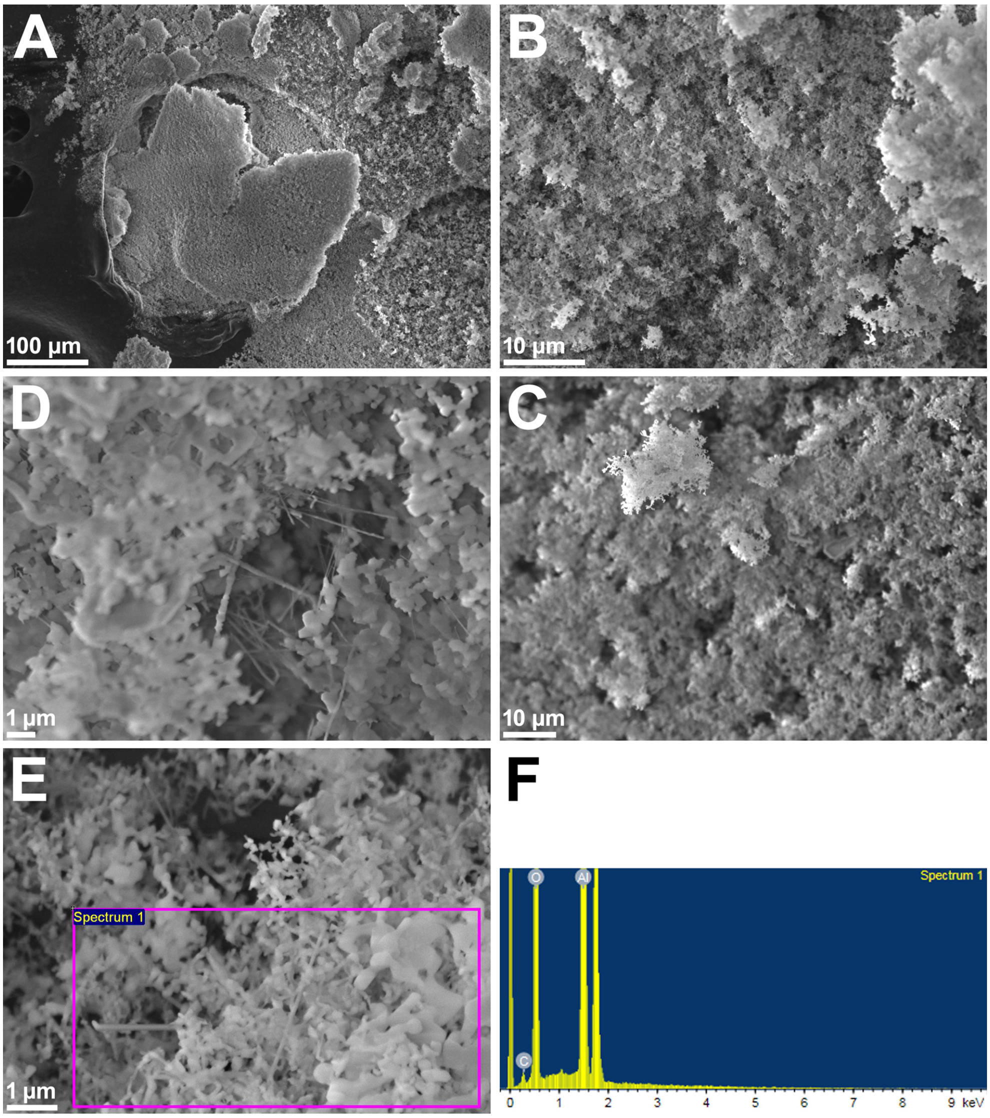

Figure 1. The SEM micrographs (**A–E**) and EDS analysis (**F**) of Al_2O_3 layer scratched from Ti_3AlC_2 pellet, deposited on the carbon tape.

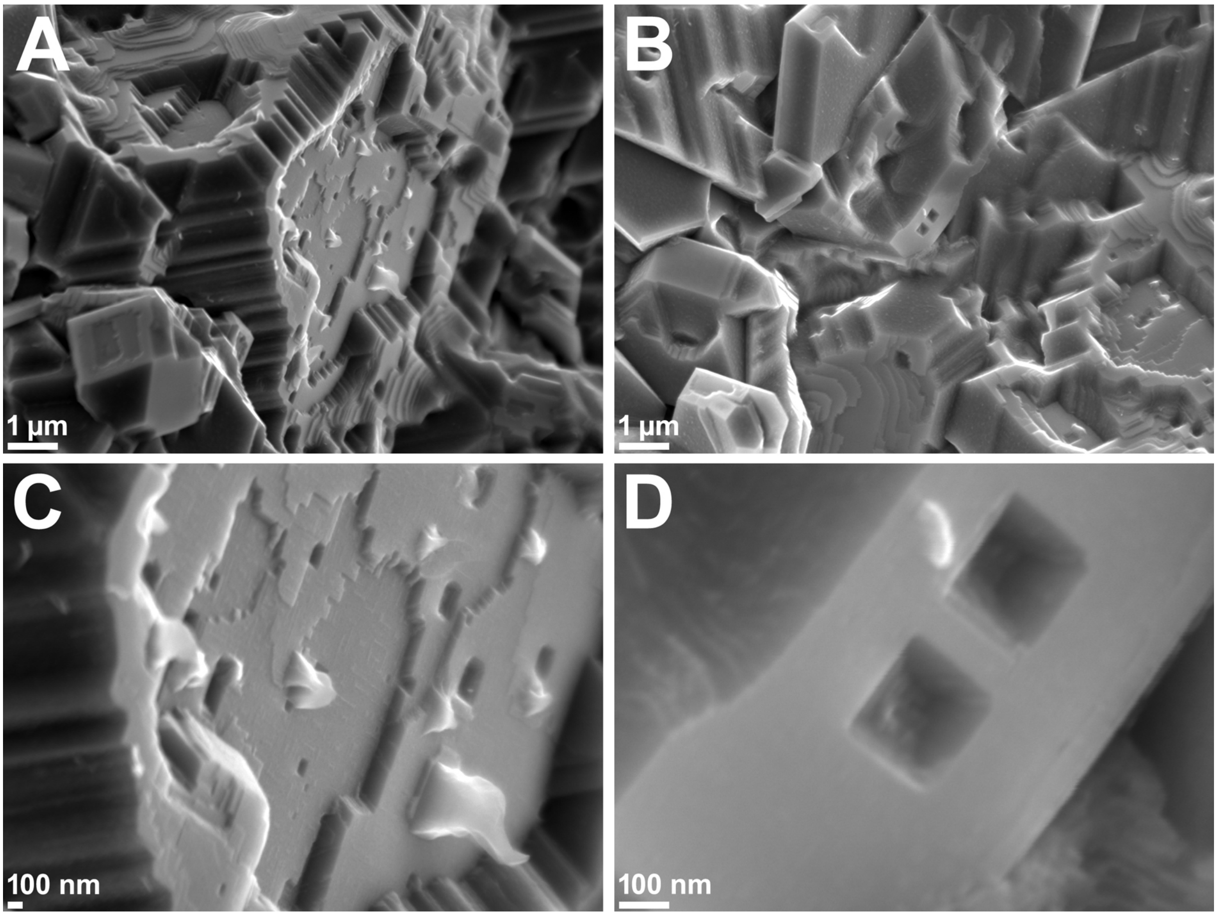

Figure 2. The SEM micrographs of Ti_3AlC_2-Air and (**A**,**C**) and Ti_3AlC_2-Ar (**B**,**D**) lightly broken pellets presenting triangle-shaped α-Al_2O_3 nanoparticles (**C**) and highly symmetrical leftover holes (**D**).
